# Evaluation of Preclinical Task Based Learning program in Medical Education

**DOI:** 10.12688/f1000research.109913.1

**Published:** 2022-03-07

**Authors:** Roopashree Shenoy, Animesh Jain, Bhagyalaksmi K, Arun Shirali, Sneha Shetty, Anand Ramakrishna

**Affiliations:** 1 Physiology, Kasturba Medical College, Mangalore, Manipal Academy of Higher Education, Manipal, Karnataka, India, Manipal, India; 2 Medical Education, Kasturba Medical College, Mangalore, Manipal Academy of Higher Education, Manipal, Karnataka, India, Manipal, India; 3 Community Medicine, Kasturba Medical College, Mangalore, Manipal Academy of Higher Education, Manipal, Karnataka, India, Manipal, India; 4 Medicine, Kasturba Medical College, Mangalore, Manipal Academy of Higher Education, Manipal, Karnataka, India, Manipal, India; 5 Respiratory medicine, Kasturba Medical College, Mangalore, Manipal Academy of Higher Education, Manipal, Karnataka, India, Manipal, India

**Keywords:** Early clinical exposure, student evaluation, feedback, Standardized patient, focus group discussion

## Abstract

The conventional curriculum in preclinical medical education has a need for early clinical exposure programs that help in correlation of basic science data with clinical skills. This is helpful to develop clinical reasoning skills, problem-solving abilities, team work, communication skills and overall attitudes and behaviour relevant for a healthcare provider. Preclinical task based learning (TskBL) is an active learning strategy in which the focus for the first year medical student is a real task done by a doctor. In this strategy the student-doctors undergo a standardized patient encounter and discuss the learning issues related to the task in the first year of medical school. The current study is focussed on the student perception of the effectiveness of task based learning module.The TskBL was conducted among first year medical students for nine topics that are commonly encountered in the clinics. After TskBL was planned and implemented the evaluation of the modules was done using focus group discussions. The students highlighted the importance of standardized patients in the TskBL strategy in providing early clinical exposure in preclinical medical education. They reported its usefulness gaining essential knowledge, skills and attitudes for medical learning. They reported positive outcomes of module design and processes and activities in TskBL. Based on the negative aspects of the modules, future improvement was suggested in improving the usefulness of standardized patient encounter. This study showed the novice learners’ outlook of the potency of TskBL for several other topics of clinical relevance to provide early clinical exposure in medical schools.

## Introduction

In preclinical education, students are mainly confined to classrooms and laboratory without any exposure to real clinical scenarios. Basic science education is crucial for medical learning but is often spent in isolation from clinical practice. ^1^ Basic knowledge and skills are unarguably important but insufficient by itself. The learners must be inspired to learn about clinical relevance and embrace medicine in all its aspects to serve patients and become the better healthcare professionals of the future. ^2^

Early Clinical Exposure (ECE) programs have paved the way for learners to get exposed to clinical learning in the first year of medical school. ^3^ The ultimate goal of ECE is to provide relevance to basic science learning, improvement of clinical knowledge, acquisition of generic transferable skills and development of better attitude as a health care provider. ^4^^–^^6^ The National Medical Commission (formerly known as the Medical Council of India) has made ECE mandatory in preclinical education. ^7^ This has led to development and implementation of a myriad of early clinical exposure strategies.

Preclinical Task based learning (TskBL) is a small group strategy that utilises a standardized patient as a trigger for providing early clinical exposure. This simulated learning approach involves the students to focus on cases that are routinely encountered in the clinics. ^8^ In TskBL students interact with the standardized patient and then discuss the learning issues relevant to the patient’s condition. In our study, a TskBL program using standardized patients was evaluated in the preclinical setting.

Evaluation of TskBL program by studying students’ perception will allow us to understand if the learning outcomes align with the design and procedures of the TskBL program. Understanding the learners perceptive will also help to scrutinize the effectiveness of processes, activities and assessment in TskBL. Knowing about the limitations will help towards strengthening the TskBL program and will allow room for improvement in planning and implementation of future modules.

Student evaluation of a preclinical task-based learning program using standardized patients has not been studied previously, this is particularly relevant in India as the national medical commission has mandated competency-based medical curriculum which includes ECE programs for medical students since 2019. The aim of this study was to evaluate the TskBL program in physiology MBBS curriculum using students focus group discussion.

## Methods

### Preclinical TskBL program

A total of nine9 TskBL module were planned and implemented. Two TskBL modules for the topic of myocardial infarction (chest pain) and myopia (blurry vision) was conducted in the academic year 2017-18. Three TskBL for the topics anemia (fatigue), hypertension (headache), and hypothyroidism (weight gain) were conducted in the year 2018-19 and four TBLs for the topics, myasthenia gravis (weakness in the evening), chronic obstructive pulmonary disease (shortness of breath), diabetes mellitus (frequent urination), upper motor neuron lesion (paralysis of right side) were conducted in the academic year 2019-20.

TskBL took place in two sessions. The first session was the standardized patient encounter and took place for the duration of one hour. The second session was discussion period which took place for the duration of two hours. Both sessions had a group which consists of 14 students, and they were guided by a faculty facilitator.

During the first session of standardized patient encounter, the team of students encountered the standardized patient. The session began with choosing a leader, a time- keeper and a scribe. The team then proceeded with history taking. Next, they performed focused physical examination or an analysed laboratory reports relevant to the patient’s history. This session ended with patient education, where the student-doctors provided information about the condition applicable to the patient. At the end of session I, the students prepared a case summary. Session I took place for the duration of one hour.

The second session took place after one week of session I. This duration is provided for the learners to prepare for discussion in second session. The student study guides containing the specific learning objectives and study material relevant to the task guided the students during this period. Session II started with students’ presentation of case summary in a sequential manner. This was followed by discussion of learning objectives. After this the learners underwent mini-quiz in which they answered the case based questions posed by the facilitator. Other similar cases were discussed This was followed by development of skills facilitated through role play. The facilitator emphasised the key points and provided a summary. Second session ended with debriefing session in which the facilitator motivated the students to reflect on their activities in session I and session II. Facilitator feedback and standardized patient feedback related to learners’ performance on history taking, clinical examination and patient education, communication skills, teamwork and their attitude as a provider of healthcare were also provided. Session II took place for the duration of two hours.

### Role of faculty

The role of faculty in TskBL session I is that of a moderator. This is identical to the role of the facilitator in problem-based learning. ^9^ The facilitator encouraged the learners to actively participate in standardised patient encounter. They also provided direction to the participants to complete all the processes in an effective and timely manner. They recorded their feedback related to history taking, physical examination and patient education.

In session II of TskBL, the facilitator motivated the students to engage in discussion and promoted the development of skills like counselling the patient to provide education through mode of role play. They highlighted the vital issues, summarised the task and discussed other similar cases. During debriefing, the facilitator encouraged the students to reflect on their own performance. They also imparted feedback to the students regarding their performance in session I and session II.

### Evaluation of TskBL

Evaluation of the TskBL program was done using focus group discussion. Focus groups are extensively used in education research to understand participants' perceptions, views and the processes underlying the ^10^ Focus groups can explain both what participants think and why they think as they do. ^11^ Participating individuals were encouraged to react to one another's opinions and form new ideas from varied perspectives.

### Procedures

This qualitative analysis was undertaken as an adjunct to a larger non-equivalent cross over study involving 750 students for three academic years (2017-18, 2018-19, 2019-20). The methods and quantitative results of this educational trial has been previously published. ^12^ The participants included in this qualitative analysis were those allocated to the educational intervention (TskBL) group.

### Sample

All the students who underwent TskBL were invited to participate in focus group discussion. A reminder was sent after one week. The students who responded were assigned to focus groups according to availability.

A total of 49 students consented to participate in 8 focus group discussions. 17 students participated in three FGDs in the academic year 2017-2018, 19 students participated in three FGDs the academic year 2018-2019 and 13 students participated in two FGDs the academic year 2019-2020.

Each focus group discussion was scheduled for one hour duration. It consisted of 5-7 students. The discussion was guided and facilitated by a moderator who was familiar with the concerned modules and was an experienced moderator of focus groups. The assistant moderator took notes and made sure the sessions were audio taped. Occasionally, they asked the participants to clarify statements whenever needed. A focus group discussion guide was prepared which contained questions relating to the following headings:

• TskBL module design
• Facilitation of TskBL
• Student experiences during TskBL
• Assessment and achievements

### Analysis of the results

All focus group sessions were audio taped and transcribed literally. The transcripts were then checked for accuracy. Data were analysed inductively using thematic analysis which is the preferred procedure acceptable for identifying and sorting shared experiential themes in qualitative data. ^13^

After the data were cleaned up, they were read repeatedly to identify themes from the focus group discussion. This was done by two investigators (Roopashree Shenoy and Sneha Shetty). They separately identified the main themes that emerged from each transcript. These investigators then compared their respective findings and developed a thematic framework based on consensus. The framework was then shared with the broader team of educators to discuss and refine it. This was followed by coding the data by theme by one investigator (RS) and key data representing each theme was extracted. Next, the sub-themes were identified and illustrative quotations were selected. These data were reviewed by another investigator (SS) to ensure these were consistent with the initial interpretation of the raw data, and then shared and discussed with a team of educators. Further refinements to the interpretations were made as a result of this process.

Consistent with other qualitative studies, a disciplined attitude was maintained in the data collection and analyses procedures. The final report was sent to all participating individuals for approval.

### Ethical considerations

The study was approved by Institutional ethics committee of Kasturba Medical College, Mangalore (IEC KMC MLR 04-17/77). Only those students who volunteered to participate were included in the study and informed consent was obtained from all the participants.

## Results

After the audio data were collected, they were transcribed. The transcripts were then checked for accuracy. After the data were cleaned up, they were read repeatedly to identify themes from the focus group discussion.

Six major themes emerged in the focus group data. They were:

• Effectiveness of structure and content of TskBL program
• Perceived long-term benefits of standardised patient exposure
• Opportunities for self-directed learning
• Perceptions on TskBL in comparison with other forms of learning encountered.
• Negative aspects of TskBL program
• Recommendations for further improvement

These themes are presented with sub-themes and quotations illustrating each of them.

### Effectiveness of structure and content of the TskBL program

This theme presents the learners’ views about which attributes of the strategy helped in best enhancing their learning, participation, soft skills and motivation. Learners generally had positive views and experiences about the structure and content of TskBL program. They appreciated how the strategy provides a valuable experience for a novice learner in the first year of medical education.

*Learning*

Students endorsed the strategy of TskBL which provided them with a rich learning experience in the beginning of medical school eduction. They reflected on the design and procedures of TskBL program like the standardised patient encounter, presentation of case summary, and tutor facilitated discussion. They reported that these learning experiences exceeded their expectations:

“I think initially we looked at a patient and when we didn’t know some of their symptoms but once we started asking more questions we came to a differential diagnosis and then came to a conclusion. It was very rewarding.”

Although they may not have learned everything about patient encounter, learners reported they had obtained a greater appreciation of applying the basic science knowledge in clinical setting

“I feel with TskBL we got to implement not just the things we learnt in theory class but the clinical aspects as well and we had to get the ideas together, provide treatment or check the clinical things. So that way we could actually work out what we learnt in class.”

The students were surprised to find out how TskBL motivated them to learn.

“So when we are studying generally the only motivation is marks in exams but that wasn’t the case here. We were actually working with the patient who needed your help. We felt responsible for our patient and I think that helps in the long run.”

*Participation*

This reflects the students’ perception about how this module encouraged them to actively participate throughout the TskBL.

“Everybody had something to add to the discussion and it was not a one- way teaching from the faculty. By seeing all of the team mates they were discussing the things they were diagnosing in their own way and I felt motivated to participate as well.”

*Experience*

This theme encapsulated how students showed their appreciation of the interactive experience and the “realness” of standardized patient encounter. The views of the participants were as follows:

“I like that we could approach the patient, take case history and look at signs and symptoms and such things and come to a conclusion. I felt like a real doctor”

“Whatever we learned we could put into practice. For the first time rather than putting what we learn on test paper or exam paper we are actually using it somewhere so that kind of give like a sense of motivation and happiness. It just makes you feel like you know something in the field.”

*Development of soft skills*

The participants unanimously opined that the TskBL approach was beneficial to develop soft skills like empathy.

“When we talked to the patient, we talk about his family and hobbies and everything so we kind of get to know the family history of the person also in a way so like that maybe he is from a poor financial background, you start empathising with the person which is more important”

*Motivation*

The participants discussed how this module motivated them and inspired them to learn more.

“I was excited when I got a patient and I think that naturally helped me learn. I was motivated enough so when I actually got a patient I had to come to a diagnosis. I felt this is why I am studying medicine and I could look at the big picture.”

The students also observed that participating in the programme helped them to understand the process in the real- world application of basic science knowledge.

“I feel if they give us more modules like this we come to know what actually happens in real clinical scenario. It was very helpful to relate what we learnt in anatomy, physiology and biochemistry to clinical application and how it is managed it in real life. Actually understanding what it feels like working in an actual atmosphere as a doctor is really helpful to motivate you.”

### Perceived long-term benefits of standardised patient exposure

This theme represents the learners’ insights about benefits of standardised patient exposure.

And how it will affect them in a positive manner in the long term.

*Correlation of basic science knowledge with clinical learning*

Overall, the students recognised TskBL to be beneficial to their learning, because it helped them in correlating basic science with clinical learning. The perceptions of the informants were as follows. The participants agreed that there is a requirement for TskBL strategy in the curriculum to provide early clinical exposure.

“In basic science classes we were reading a lot of theory and practising clinical skills on each other but with this module I understood that the patient isn’t a textbook or worksheet that you can fill out and answer. I felt like I could apply what I’ve learnt.”

"I believe that physiology practicals had different tests and clinical exams but we never knew how to connect them with the patient. Here we got the specific order in which we have to follow and when we had the discussion, we understood what we did was correct or wrong and how we could do it better so that very effective for me and I still remember the particulars of how to exactly approach a patient.”

*Development of clinical and diagnostic reasoning*

The students discussed how this strategy encouraged them to develop clinical and diagnostic reasoning throughout the TskBL and how it influences their professional future.

“We realised that and we have to think about all aspects of patients life to actually diagnose their condition. Even when we write patient particulars like name age, sex and occupation all of that is really important to come to a diagnosis.”

Specifically, they felt that the facilitator helped them to relate the application of knowledge to the patient scenario.

“The tutor encouraged us to take more details of the patient. She asked us whether this information that we've asked and taken down is enough. when the patient said that he had a pain in his chest we always thought about what we learned and that is MI and then she pushed us like do want to think of something else like probably something to do with the lungs… And another system? And she helped us to relate what we already had learnt theory to apply to the patient encounter.”

*Development of communication skills*

The students perceived standardized patient encounters to be useful to boost the development of communication skills.

“I feel we understood what the patient expects from us. For example: we had used some medical terms which he didn't understand he expected us to put it in simple language and make him understand. So I learnt that as the patient is not of a medical profession or doesn’t have medical knowledge so the test we did or performed on him we should let him know what kind of test we want to do and what are the procedures how he can help us that we may get a better diagnosis.”

Furthermore, the participants pointed how TskBL helped in interpersonal communication

"In case of any confusion or if we are having any problem with the diagnosis when we discussed among our team we got to learn many things and the way we have to patiently listen to them we have to think about their aspect of thinking in that particular point. It is helpful. So we could this in different perspective as well because we were working in a team.”

*Retention of knowledge and skills*

All students unanimously agreed that approach was beneficial to retain the knowledge and skills learnt. The views of the participants were as follows:

“I feel at the end of the day TskBL is a sort of a practical based learning. So it will always be better than learning in the classroom and I think we will be able to remember it better.”

### Opportunities for self-directed learning

This theme represents the students’ views about how TskBL provided them with opportunities for self-directed learning. The following narratives revealed these perceptions:

“I feel the study guides really helped me to learn on my own. A guide would provide you with more information and when we learn from it, it will stay more in our head than when we hear from another person.”

Furthermore, the participants elaborated on how they were influenced by the standardized patients to be responsible physicians.

“I feel in TskBL we have to study in-depth because we explain to a patient and we cannot give them wrong information so we look deeply into the topic that kind of helps us because we have a broad view of what’s happening even if it is a small topic. We learn on our own in such a way that we don’t have to look back at it when exam comes.”

### Perceptions on TskBL in comparison with other forms of learning encountered

This theme represents the students’ views about how TskBL compared to other forms of learning they have encountered. Students generally had positive perceptions about TskBL when compared to other forms of learning. They liked the interactive nature of the modules. Besides, they also felt that the strategy was very different when compared to other traditional small group teaching strategies like problem-based learning, team- based learning and tutorials that are routinely implemented in the preclinical curriculum.

*Problem-based learning (PBL)*

The students appreciated how TskBL and PBL can supplement each other in the first- year medical curriculum to boost learning. The views of the participants were as follows:

“I Feel both TskBL and PBL are equally important because PBL helps us to understand why that patient has a particular issue and we need to know that that to actually treat the patient in the clinics. TskBL will help us with how we treat the patient like when we get an actual patient and not just a case on paper like in PBL. So both are important.”

“In PBL we are actually analysing the whole story that happened that the patient had a disease. Just making note of them we are extracting the points to learn. But in TskBL you are actually applying the practical knowledge as well as the theoretical knowledge. Your try to make your diagnosis as much accurate as possible. You need to have lot of knowledge for that and you discuss but in PBL, we just have to find the objectives and present it. In this TskBL everything is done plus the practical skill is also used.”

*Team based learning (TBL)*

The students compared their experiences in TskBL and TBL strategies and how they were benefitted from both educational interventions.

“TBL made us to think about real world application of basic science using problems and TskBL made us to learn using standardized patients. I really benefited from both.”

*Tutorial learning*

The students had positive perceptions about TskBL when compared to tutorial learning. The following narratives showed these perceptions:

“I felt that more people were active in TskBL Like during tutorial many people don’t get involved in it totally but here like everybody had to give some input and they did, so I felt it was better.”

### Negative aspects of TskBL program”

While positive opinions and beneficial effects of TskBL were mentioned by the learners they also presented with comments on the negative attributes of the TskBL strategy.

*Large group size*

The students discussed how this the large number of students for one standardized patient hampered their learning in TskBL. Some examples can be seen in the following narratives:

“We involved many people participating together which puts a pressure on missing out on some tasks or clinical examinations by some students. If the TskBL was designed in such a way that only target the small number of students at a time and that might have been helpful.”

*Intense standardized patient encounter experience*

The students felt that standardized patient encounter can be challenging for a first- year student of medicine. Their views are as follows:

• “I think in the beginning to get that connect with the patient was difficult as we are facing a patient for the first time ever, it was a little overwhelming at first but it was fine when we knew how to proceed with it as a team.”

### Recommendations for further improvement

With respect to the negative aspects of the TskBL strategy, there were several recommendations for improvement. This included working in smaller group. Some examples can be seen in the following narratives:

• “I feel we should work in smaller group of 5-6 or 2-3 students instead if 14 students in one group. That way participation and learning can be better.”

The students also suggested a chronology which can be followed while conducting ECE modules so that the novice learner doesn’t get overwhelmed by intense experience during patient encounter.

“Just how you are allowing us to meet standardised patient in first year before meeting the real patient in the clinics next year. We could receive modules like PBL and TBL much before we undergo TskBL. In that way we are not confused or overwhelmed by standardized patient encounter.”

Furthermore, students wished to receive more TskBL experiences during their training

• “I feel we should have more modules of this nature with different type of standardised patients for example children. If a person want to pursue paediatrics then a TskBL involving child patient will be beneficial, then the way you treat that patient will be different from the way you treat an adult. So I guess that way you can get more exposure and you actually understand what you are getting into.”

Some recommendations on overcoming language barrier by introducing language classes were made.

• “I think we had language barrier because we didn’t know the regional language that he patient spoke. We have to overcome this language issue and learn the local language. Classes on local languages should happen in medical training because ultimately we interact with patients and it is not possible if we don’t know the language.”

## Discussion

**Figure 1.**
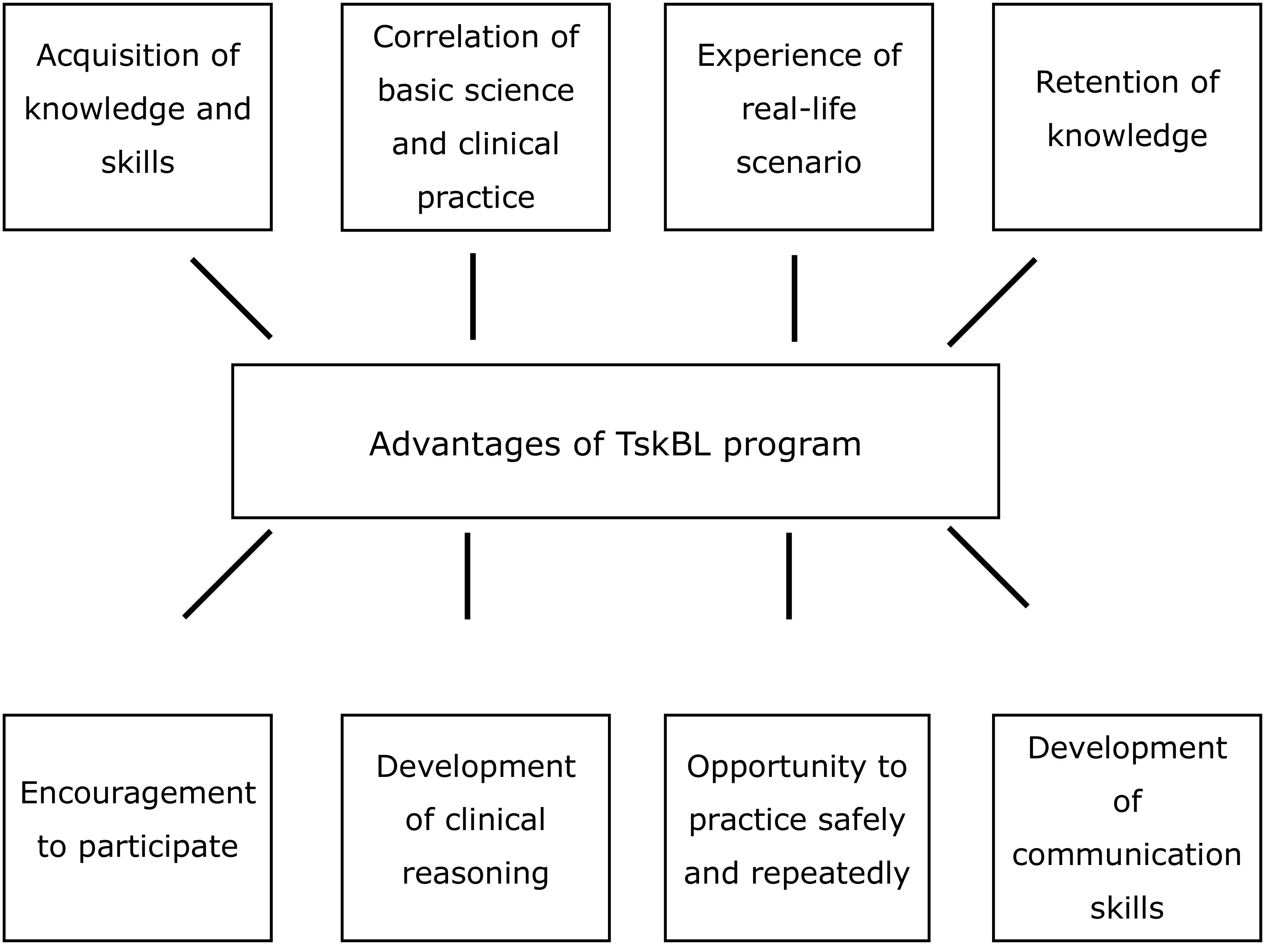
Advantages of preclinical TskBL.

Employing the focus group discussion method, this work evaluated the outlook and experiences of students who participated in the task- based learning program. The students generally had a positive opinion about the program, what they had achieved and the program’s relevance to their career in health care. Figure 1 gives the advantages of TskBL program. The learners' request to receive more TskBL experiences throughout their study period as a student-doctor strengthened these findings. Many lucid strengths, weakness and future recommendations also emerged.

Based on thematic analyses, various observations were highlighted from the results obtained. The students perceived that there were many positive attributes of the modules. This included promotion of active learning, application of basic science in the clinics and development of various skills and attitudes that are required in a healthcare career. These findings are similar to the results highlighted in the previous studies conducted during the clinical years of medical education. They showed the importance of TskBL program in providing motivation to learn, improving learners’ knowledge and performance skills and boosting their all- round development. ^14^^,^^15^ The learners explained how TskBL strategy was helpful to co-relate theory with practice and to understand the relevance of basic science knowledge in clinical learning. This strategy also helped in understanding the importance of empathetic doctor-patient interaction. Most of these gains were attributed to the presence of standardized patient. The simulated patient encounter was highlighted as the crucial component that provided motivation for further learning. This may be because standardized patient encounter is known to enhance performance in diagnostic reasoning, clinical exposure and essential skills required in the preclinical curriculum. ^16^^–^^18^

The learners appreciated the interactive nature of the educational strategy. They informed that it helped them to develop their communication skills in two ways. First, it helped them to interact with the patient in an efficient manner. They learnt that patient is a common man who would not understand “medical speak” and tried to incorporate words which was understood easily. Second, they commented about how TskBL helped in interpersonal communication. A previous study conducted in a medical university in Malaysia also concluded that TskBL helped in development of acquired skills such as interpersonal communication. ^19^

The participants perceived TskBL to supplement the existing medical curriculum and opined that it could be helpful alongside problem-based learning and team- based learning to provide early clinical exposure. They compared the standardized patients in TskBL to the paper scenario in PBL and concluded that PBL helped them to think in an analytical manner while TskBL boosted the development of skills as well in addition to applying the knowledge to the clinical setting. This is supported by the findings of clinical TskBL in Dundee medical curriculum where TskBL was used as a basis for integration and problem-based learning. ^20^ In comparison with team- based learning, the students voiced their request to experience both the modules as they had benefitted from the real-world application of basic science in both TBL and TskBL.

However, the students also perceived negative attributes of the strategy. The most common negative aspect of TskBL strategy was that this strategy employed large number of students per standardized patient. TskBL had 14 students, 1 standardized patient and 1 facilitator in a group. The groups were formed in this manner because of two reasons. One, our educational set-up consisted of large student population of 250 students per academic year. Dividing the students into groups smaller than fourteen would require additional time, effort and resources. Second, in our educational institution the students were allotted into groups of 14 or 28 for all the small group learning activities in the physiology course. Bypassing any or both of the above-mentioned reasons was not feasible in our study setting. The other negative experiences of TskBL strategy were identified as being too ‘intense’ and ‘overwhelming’ for a first- year medical student.

Based on the negative attribute of the TskBL modules, there were several suggestions for further improvement. This included increasing the effectiveness of standardized patient encounter by working in small group of students per standardised patient. This could be because of increased opportunity to interact with the standardized patient, individual participation in all the procedures of the standardized patient encounter including history taking, clinical exam and patient eduction and higher attention from the faculty facilitator. All of this could lead to the possibility of having a better learning experience if fewer students were recruited per standardized patient. ^8^^,^^15^^,^^21^

The students also opined that they wished to undergo many more modules of this nature throughout their medical learning with different type of standardised patients (for example children). They also had recommendations on overcoming language barrier by introducing language learning classes for medical students to perform better patient interaction in the clinics.

Despite the fact that the current findings are quite positive, there were alternative explanations. The student participant may play the role of a ‘model informer’ by providing only positive point of view about the TskBL strategy. However, this was unlikely because the participants were encouraged to express their views and negative outlook openly and they were assured that they could communicate the negative comments about the modules. Another explanation for the positive findings could be that the focus group dynamic moulds the agreement of the opinions involved. However, this possibility was low, because the focus group interviewer was an experienced moderator of focus groups with significant experience in conducting focus group discussions.

Another limitation of the study is that the current study does not capture the complete evaluation of the TskBL module. This is because it does not discuss the faculty perception about the TskBL strategy. Obtaining feedback from the faculty who were involved in planning, implementing and assessing the TskBL modules would be a valuable addition to a comprehensive evaluation of the TskBL strategy.

The present study highlights the importance of TskBL strategy as perceived by students in providing early clinical exposure in preclinical medical education and despite the drawbacks, the present qualitative findings substantiate the positive aspects of TskBL strategy and further confirm its effectiveness in boosting the overall development of preclinical students.

## Data availability

### Underlying data

Open Science Framework. task based learning. DOI: https://doi.org/10.17605/OSF.IO/3B4NU ^22^

This project contains the following underlying data:

- Interview recordings: All the students who underwent TskBL were invited to participate in focus group discussion. A reminder was sent after one week. The students who responded were assigned to focus groups according to availability. Each focus group discussion was scheduled for one hour duration. It consisted of 5-7 students. The discussion was guided and facilitated by a moderator who was familiar with the concerned modules and was an experienced moderator of focus groups. The assistant moderator took notes and made sure the sessions were audio taped. Occasionally, they asked the participants to clarify statements whenever needed. A focus group discussion guide was prepared which contained questions relating to the following headings: TskBL module design Facilitation of TskBL Student experiences during TskBL Assessment and Achievements.

Data are available under the terms of the Creative Commons Zero “No rights reserved” data waiver (CC0 1.0 Public domain dedication).
