# Supplementary material for: Evaluation of Preclinical Task Based Learning program in Medical Education
Source: F1000Res. 2022 Mar 7;11:282. [Version 1] doi: 10.12688/f1000research.109913.1 (PMC10521084; doi:10.12688/f1000research.109913.1)
Supplement: Peer review history

# Reviewer report: Reviewer response for version 1

**DOI:** 10.5256/f1000research.121469.r195911

**Published:** 2023-09-15

**Authors:** Bulan Kakanita Hermasari

**Affiliations:** 1 Sebelas Maret University, Surakarta, Indonesia

The article writing is clear and accurate, yet it contains several grammatical issues that need to be read through. The number of references is 24 articles, but almost two-thirds of them are not updated articles (before 2013).

 This article does not provide explanation of trusthworthiness especially regarding triangulation. In the methods section, the authors mentioned about the educational trial that has been previously published. For the aim of triangulation and generalizibility of the study, the authors could explain more about these qualitative results and its relation with the quantitative finding.

Is the work clearly and accurately presented and does it cite the current literature?

Partly

If applicable, is the statistical analysis and its interpretation appropriate?

Partly

Are all the source data underlying the results available to ensure full reproducibility?

Yes

Is the study design appropriate and is the work technically sound?

Yes

Are the conclusions drawn adequately supported by the results?

Partly

Are sufficient details of methods and analysis provided to allow replication by others?

Yes

Reviewer Expertise:

medical education; interprofessional education and practice; reflective learning; assessment in medical and health profession education; professionalism

I confirm that I have read this submission and believe that I have an appropriate level of expertise to confirm that it is of an acceptable scientific standard, however I have significant reservations, as outlined above.

# Reviewer report: Reviewer response for version 1

**DOI:** 10.5256/f1000research.121469.r140791

**Published:** 2023-09-15

**Authors:** Damodara Gowda K M

**Affiliations:** 1 Department of Physiology, K.S. Hegde Medical Academy, Nitte (Deemed to be University), Mangaluru, Karnataka, India

As it was rightly mentioned in the article that in preclinical education, students are mainly confined to classrooms and laboratories without any exposure to real clinical scenarios. Basic science education. This is the basic need of the I Prof students. Therefore, NMC has introduced early clinical exposure in the I Phase. The thematic approach in the present study is apt. The short forms of investigators SS and RS might have been spelled in their first appearance.

Is the work clearly and accurately presented and does it cite the current literature?

Yes

If applicable, is the statistical analysis and its interpretation appropriate?

Yes

Are all the source data underlying the results available to ensure full reproducibility?

Yes

Is the study design appropriate and is the work technically sound?

Yes

Are the conclusions drawn adequately supported by the results?

Yes

Are sufficient details of methods and analysis provided to allow replication by others?

Yes

Reviewer Expertise:

Neurobiology, Molecular aspects of cognition

I confirm that I have read this submission and believe that I have an appropriate level of expertise to confirm that it is of an acceptable scientific standard.

# Reviewer report: Reviewer response for version 1

**DOI:** 10.5256/f1000research.121469.r126481

**Published:** 2022-05-06

**Authors:** Somsubhra De

**Affiliations:** 1 Department of Obstetrics and Gynecology, Melaka Manipal Medical College (MAHE), Malacca, Malaysia

Incorporating a clinical approach in first-year helps in better understanding of the subject conceptually. However, variation of difficulty in the modules might lead to bias in perception and the authors should comment on this **.**

 The authors may want to think of practical solutions for the feasibility issues alongside addressing how these students learnt history taking and examination skills in first-year.

 In the methods, I saw the First Year students were taking history and doing physical examinations for the standardized patient. Usually, First Years are not routinely trained for History taking or clinical/physical examination on standardized/simulated/real patients in detail. It is as mentioned rightly, they are usually in a confined environment or lab setting. So, I was wondering how these students were successful in doing so. Maybe the authors did facilitate the process or gave them a briefing/short training about those skills prior to the study. The authors may clarify by stating either of the options in my previous sentence.

 Acronyms need to be standardized eg TskBL /TBL in methods, RS SS in Analysis of Results

Is the work clearly and accurately presented and does it cite the current literature?

Yes

If applicable, is the statistical analysis and its interpretation appropriate?

Not applicable

Are all the source data underlying the results available to ensure full reproducibility?

Yes

Is the study design appropriate and is the work technically sound?

Partly

Are the conclusions drawn adequately supported by the results?

Yes

Are sufficient details of methods and analysis provided to allow replication by others?

Partly

Reviewer Expertise:

medical education and interprofessional education

I confirm that I have read this submission and believe that I have an appropriate level of expertise to confirm that it is of an acceptable scientific standard, however I have significant reservations, as outlined above.
